# Physical exercise, IGF-1 and cognition A systematic review of experimental studies in the elderly

**DOI:** 10.1590/1980-57642018dn12-020003

**Published:** 2018

**Authors:** Angelica Miki Stein, Thays Martins Vital Silva, Flávia Gomes de Melo Coelho, Franciel José Arantes, José Luiz Riani Costa, Elizabeth Teodoro, Ruth Ferreira Santos-Galduróz

**Affiliations:** 1 Institute of Biosciences, UNESP (Universidade Estadual Paulista) Physical Activity and Aging Lab (LAFE), Rio Claro, SP, Brazil.; 2 Instituto Federal Goiano - Campus Morrinhos, Morrinhos, GO, Brazil.; 3 Postgraduate Program in Physical Education, Federal University of Triangulo Mineiro, Uberaba, MG, Brazil.; 4 Center of Mathematics, Computing and Cognition, UFABC, University Federal of ABC, Santo André, SP, Brazil.

**Keywords:** cognition, exercise, older adults, cognição, exercício, adultos idosos

## Abstract

One of hypothetical mechanisms related to cognition is exercise-induced IGF-1.

**Objective:**

The aim of this study was to analyze the effects of exercise on IGF-1 levels and cognition in the elderly.

**Methods:**

The article searches were conducted on Pubmed, Web of Science, PsycINFO and Scielo databases and reviewed according to PRISMA guidelines. The inclusion criteria were: [1] original articles published up to 2017; [2] samples including elderly; [3] protocols including physical exercise; [4] longitudinal studies having exercise as main outcome; [5] assessment of IGF-1; [6] cognition assessment.

**Results:**

Seven studies were included in this review. Three of the studies showed an exercise-induced increase in IGF-1; three found stable IGF-1 levels and one found a reduction in IGF-1; with and without improvement in cognition.

**Conclusion:**

Disparities in the type of physical exercise, protocols and samples under different conditions hinder the establishment of a consensus on IGF-1, cognition and physical exercise.

## RESUMO.

Um dos mecanismos hipotéticos relacionados a cognição é o IGF-1 induzido pelo exercício.

**Objetivo:**

O objetivo deste estudo foi analisar os efeitos do exercício físico nos níveis de IGF-1 e na cognição de idosos.

**Métodos:**

A busca de artigos foi conduzida nas bases de dados Pubmed, Web of Science, PsycINFO e Scielo, revisada de acordo com as recomendações do PRISMA. O critério de inclusão adotado foi: [1] artigos originais publicados até 2017; [2] amostras incluindo idosos; [3] protocolos que incluíssem exercício físico; [4] ensaios randomizados, não randomizados, controlados e não controlados; [5] avaliação dos níveis de IGF-1; [6] avaliação cognitive.

**Resultados:**

Sete estudos foram incluídos nesta revisão. Três deles mostraram aumento de IGF-1 por meio do exercício; três verificaram manutenção nos níveis de IGF-1 e um, verificou redução de IGF-1; com e sem melhora cognitive.

**Conclusão:**

Diferenças nos tipos de exercício, protocolos, amostras em diferentes condições dificultam o estabelecimento de um consenso em relação ao IGF-1, cognição e exercício físico.

The confluence of studies in animals and humans about the benefits of physical activity on brain health has attracted attention to the creation of interventions, in the form of programs that are able to promote and protect brain health and prevent diseases in the elderly population.^1^^,^ 2 A previous study highlighted some neurophysiological hypotheses in which physical exercise is able to promote benefits in brain health, such as the regulation of reactive oxygen species, growth factor release, neurotransmitter synthesis, brain oxygenation increase, glucose uptake and changes in cerebral blood flow.^3^ Therefore, one of the feasible underlying factors associated with cognitive improvement due to exercise is growth factor. Some evidence suggests that physical activity and physical exercise influence the brain through circulating growth factors, which cross the blood barrier and modulate several mechanisms for cognition.^4^ Among these factors, brain-derived neurotrophic factor (BDNF), insulin-like growth factor-1 (IGF-1) and vascular endothelial growth factor (VEGF) have been indicated as the main factors, since they work in conjunction to produce functional effects related to plasticity, functioning and brain health.^5^ In this context, IGF-1 seems to be a mediator of exercise effects on brain health, since it appears to regulate BDNF and VEGF,^5^ protecting against injuries to the brain, while improving memory and spatial learning cognitive functions.^6^

Peripheral IGF-1 levels are quickly increased in humans in response to physical exercise.^7^ This increase seems to play an essential role for exercise-induced neurogenesis^8^ and memory improvement,^5^ being a feasible moderator in response to exercise related to BDNF and cognitive performance.^9^ In a previous systematic review, the relationship between physical exercise (moderate aerobic exercise intensity) was observed, which increased BDNF peripheral levels and cognitive improvement in healthy elderly and elderly with different pathologies.^10^ Some of the analyzed papers in the review, in addition to the BDNF levels found, IGF-1 evaluation was included after a training period, showing cognitive improvement^11^^,^ 12 and increased peripheral IGF-1 levels.^12^

In studies with elderly, high serum IGF-1 levels were associated with better cognitive performance^13^ while for elderly with cognitive impairment, low IGF-1 levels were associated with poor cognitive performance.^14^ Moreover, circulating IGF-1 levels seemed to be associated with cognitive performance in the elderly, where the hippocampus appeared to be a primary target for IGF-1.^15^ On the other hand, irrespective of peripheral increase, there was an exercise-induced increase in the hippocampus with improvement in cognitive performance.^6^ Although the relationship among physical exercise, cognitive performance and IGF-1 remains unclear, it seems that physical exercise improves cognitive function and regulates IGF-1 levels, but through different mechanisms.

Most IGF-1 secretion is performed by the liver, but IGF-1 can be expressed by virtually all cell types.^16^ Also, IGF-1 can be synthesized by endocrine, paracrine and autocrine mechanisms.^17^ In relation to the nervous system, multiple effects have been attributed to IGF-1, such as neuronal signaling, neurotrophic mechanisms, neuroprotection and even pro-neuroinflammatory conditions.^18^

Although the area that IGF-1 acts on in the brain has yet to be elucidated, a study carried out by Trejo et al.^8^ indicated that blocking the entrance of IGF-1 in the brain resulted in prevention of neuronal proliferation in the dentate gyrus, reinforcing the role of this factor in neurogenesis.^9^ In addition, IGF-1 blocked receptors in choroid plexus, triggering a series of disorders such as amyloidosis, cognitive deficits, loss of synaptic vesicle protein, glucose and abnormal forms of tau protein.^19^ These disorders are similar to those found in Alzheimer’s Disease (AD).^9^^,^ 19 Therefore, public health systems should look to physical exercise programs to prevent dementia in the elderly population,^20^ given the elderly represents a large contingent of the total population in most countries. Accordingly, it is necessary to investigate the relationship between exercise-induced IGF-1 and cognition in human samples, since the clinical field requires resources to treat and prevent dementia and improve health, especially during aging. Thus, the aim of the present study was to analyze studies that investigated the effects of physical exercise on IGF-1 levels and cognitive performance in elderly.

## METHODS

### Search strategy

This systematic review was designed and developed according to the Preferred Reporting Items for Systematic reviews and Meta-Analyses (PRISMA).^21^ The methodological plan of this study was to search and analyze studies that investigated the effects of physical exercise on the concentration of IGF-1 and cognitive function in the elderly. The bibliographic search was conducted on the following databases: Pubmed, Web of Science, PsycINFO and Scielo, including articles published up to 2017.

The keywords and boolean operators were: “physical exercise” OR “physical activity” OR “physical therapy” OR “exercise” OR “training” OR “fitness” AND “cognitive functions” OR “cognitive” OR “cognition” AND “Insulin-like growth factor 1” OR “IGF-1” AND “elderly” OR “older” OR “aged”.

After the search, the articles were analyzed according to the following: [1] Title analysis; [2] Abstract analysis; [3] Full-text analysis of each article; [4] Article selection. In addition to the search on the databases, a manual search in the reference list of the selected papers was carried out.

### Inclusion criteria

For the selected articles, some criteria were adopted: [1] original articles published up to 2017; [2] sample including elderly population; [3] protocols including physical exercise; [4] longitudinal studies having exercise as main outcome; [5] Assessment of circulating IGF-1 levels; [6] Cognition assessment.

### Studies selection

The whole process of selecting papers was conducted by 2 evaluators (AMS, TMVS), that fulfilled all the steps for paper selection - from the title to abstract and article analysis. In the event of disagreement between the evaluators over article inclusion, a meeting was held to make a decision. All the articles were reviewed in May of 2017. In addition, a registration was made in all databases in case new articles with the same keywords emerged, so that notification messages would be sent to the evaluators.

## RESULTS

**Figure 1:**
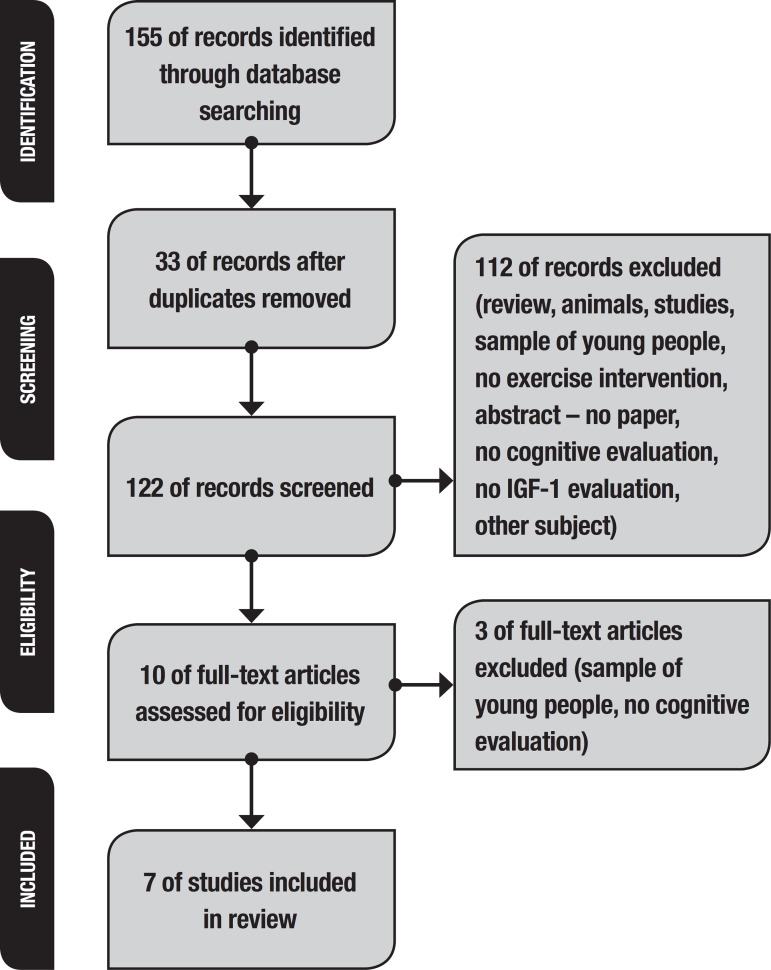
The PRISMA flowchart of the systematic review.

The search using the mentioned keywords led to the retrieval of 155 articles. In initial screening, 41 articles were selected by their titles. The articles were then analyzed through their abstracts, of which 31 were excluded, giving 10 articles for full-text read. For this systematic review, 7 articles were selected. From May 2017 up to the present time, no new studies, according to the “notification messages” on the databases, have been included, since the new articles were not related to the current subject. Figure 1 depicts the steps of the article selection process:

Figure 1 provides information on the selected articles used in this review.

## DISCUSSION

The aim of this review was to analyze studies that investigated the effects of physical exercise on IGF-1 concentrations and cognitive functions in the elderly. Of the 145 articles found on the databases, seven met the inclusion criteria. This is the first study on the relationship among IGF-1 levels, cognition and physical exercise in the elderly. Other reviews about IGF-1 levels and cognition have been published, but not including the effects of exercise on these variables.^22^^,^ 23

Out of the seven articles found in the present study, three found an increase in IGF-1 concentrations with cognitive improvement;^11^^,^ 24 ^,^ 25 another three found maintenance for this growth factor with and without cognitive improvement;^12^^,^ 26 ^,^ 27 while the remaining study found a decrease in IGF-1 without improvement in cognition.^28^ The same article observed that changes in IGF-1 levels were correlated with changes in functional connectivity.^28^ Maass et al. (2016)^27^ found similar results, identifying a relationship among changes in IGF-1 with hippocampal volume, hippocampal head volume and in the delayed verbal learning recall and memory test. The discussion was divided under sub-headings in order to discuss different points related to the studies.

### Samples and different responses

Baker et al.^11^ and Baker et al.^12^ employed the same aerobic training and experimental design but different samples and measurements. Similar results were found related to cognition improvement in both studies, but not associated with IGF-1 levels. In this case, the differences were probably related to the population because the training protocol, IGF-1 quantification and cognitive tests were the same. Despite the absence of IGF-1 change in one of them, the two studies had clinical relevance because both samples presented an AD risk-factor. Thus, the positive results on cognition reinforced the role of aerobic exercise as a pretreatment in this disease.^1^^,^ 29 In a second instance, besides the difference between samples, there was a difference according to gender. Therefore, the changes in IGF-1 levels were gender-dependent.^12^^,^ 25 For healthy elderly men, the study indicated that serum IGF-1 seemed to be associated with working memory, selective attention and executive function.^30^

**Table 1:** Characteristics of studies analyzing the effects of physical exercise on IGF-1 levels and cognition in the elderly.

Study (year)		Sample		Type of Exercise; Training variables		Intensity; Time/ Session; Frequency/ Week; Duration		Load		IGF-1 measurement; Quantified by		Cognition measurement (test)		Results
Cassilhas et al.^24^(2007)		62 male elderly (aged 65 to 75 years) in 3 different groups: Control group, Moderate intensity group and High intensity group		Resistance training; 2 sets of 8 repetitions, 90 seconds between sets for chest press, leg press, vertical traction, abdominal crunch, leg curls, lower back exercises.		50% of 1RM or 80% of 1RM; 60 min; 3 times; 24 weeks		Load adjustment on week 10, 15, 18 and 21.		IGF-1 serum concentration; Immunoradiometric assay.		Wechsler adult intelligence scale III - WAIS III; Wechsler memory scale-revised (WSM-R); Toulouse-Pieron’s concentration attention test; Rey-Osterrieth complex figure.		Increased IGF-1 levels and cognitive performance improvement on all tests for Moderate intensity group and High intensity group.
Baker et al.^11^(2010)		28 prediabetic elderly (mean age of 70 years), randomized to: Control group and Aerobic group		Aerobic training; treadmill, stationary bicycle or elliptical trainer		75-85% of heart rate reserve; 45-60 min; 4 times; 24 weeks		NR		IGF-1 plasma concentration; Radioimmunoassay		Trail-making test (Trail A and B); Task Switching; Stroop Color-Word Interference; Self-Ordered Pointing Test; Verbal fluency; Story Recall; List Learning.		Maintained IGF-1 levels and cognitive performance improvement in aerobic group. Improvement on: Trail B; Task switching; Stroop color-word interference and trends in Self-Ordered Pointing test and Verbal Fluency.
Baker et al.^12^(2010)		33 elderly (mean age of 70 years) with amnestic mild cognitive impairment, randomized to: Control group and Aerobic group.		Aerobic training; treadmill, stationary bicycle or elliptical trainer		75-85% of heart rate reserve; 45-60 min; 4 times; 24 weeks		NR		IGF-1 plasma concentration; Radioimmunoassay		Trail-making test (Trail A and B); Stroop color and Word test; Task Switching; Verbal fluency; Symbol digit modalities; Story Recall; List Learning; Delayed-Match-To-Sample.		Increased IGF-1 levels for men in the Aerobic group and improvement in cognition for women and men (Trail B, Stroop color and word test; Task Switching; Verbal fluency, Symbol-digit modalities), with different responses according to sex.
Voss et al.^28^(2013)		65 elderly (mean age of 67.3 years), randomized to: Control group and Aerobic group		Aerobic training; walking on an indoor track		60-75% of the maximum heart rate reserve, for the most part; 10-40 min; 3 times; 48 weeks		Increment of 5 minutes per session until week 7		IGF-1 serum concentration; enzyme-linked immunosorbent assay		Structural MRI; Functional MRI		Reduction in IGF-1 levels after intervention. Change in IGF-1 levels were correlated with increase in connectivity between the bilateral parahippocampus and the bilateral middle temporal gyrus.
Tsai et al.^25^(2015)		48 male elderly (mean age of 71.4 ± 3.79 years), randomized to: Control group and Exercise group.		Resistance training; 3 sets of 10 repetitions, 90 seconds between sets for biceps, curls, leg press, triceps extensions, hamstring curls, latissimus dorsi pull-downs, calf raises, and seated rowing.		75-80% 1 RM; 60 min; 3 times; 48 weeks		Individual load without a fixed period		IGF-1 serum concentration; chemiluminescence immunoassay		Oddball task with event-related potential (ERP) – P3a and P3b		Increased IGF-1 levels and cognitive performance improvement in the Exercise group, in Accuracy rate under the oddball condition, reaction time, P3a larger amplitude and trends for P3b.
Rahe et al.^26^(2015)		68 elderly (range 50-85 years), randomized to: Cognitive training group; Cognitive training with Physical Activity group and Cognitive training + Physical activity + Counseling		Multimodal training with cognitive training; Multimodal training with cognitive training + Counseling		NR; 20 min; 2 times; 7 weeks		NR		IGF-1 serum concentration; Chemiluminescence immunoassay and sandwich-chemiluminescent immunoassay.		DemTect.; Complex figure test; Brief test of attention; German WAIS III; Regensburger wort flüssigkeits-test; Stroop test; Key search.		Maintained IGF-1 levels in all groups. Increase in cognitive performance in both groups.
Maass et al.^27^(2016)		40 elderly (mean age of 68.4 years), pseudo- randomized to: Control group and Aerobic group.		Aerobic training, treadmill;		65% target heart rate; 30 min; 3 times; 1 2 weeks		Increment of 5% target heart rate for 4 weeks		IGF-1 serum concentration; enzyme-linked immunosorbent assay		Verbal learning and Memory test; Rey auditory verbal learning test; Complex figure test; Digit Span Test; Magnetic resonance imaging – high-resolution region-of-interest (ROI)-based and voxel-based morphometry (VBM).		Maintained IGF-1 levels and cognitive performance in the Aerobic group. IGF-1 levels were related to hippocampal volume changes (measured by voxel based-morphometry) and delayed verbal recall performance.

NR: not reported.

In this review, only two studies were exclusively about gender.^24^^,^ 25 Cassilhas et al.^24^ and Tsai et al.^25^ included only elderly men and used resistance training at 50% and 80% of 1 RM; 75-80% 1 RM, respectively, three times per week for 24 and 48 weeks. The levels of IGF-1 increased in the resistance training group, whereas in the control group, the levels were maintained or unaltered. In all cognitive functions evaluated, Cassilhas et al.^24^ found better performance compared to the control group (central executive, short-term visual modality, attention, short-term, long-term and episodic memory). The study of Tsai et al.^25^ also found improvements in cognitive function, specifically executive function and attention. Also, significant changes in accuracy rate, reaction time, and P3a amplitude from event-related potential in an oddball task - a cognitive test paradigm, were observed. Cassilhas et al.^24^ and Tsai et al.^25^ hypothesized positive results on cognition modulated by increasing IGF-1 levels. Besides, the sample was composed exclusively of men and the control group of Tsai et al.^25^ did not do any regular and systematic physical activity and showed no changes in cognitive functions or in any other electrophysiological parameter. Another point related to the sample and groups was the frequency in the Training/Exercise group and control group.

Other observations regarding the experimental design of the studies, generated restrictions related to IGF-1 levels, physical exercise and cognitive functions. Cassilhas et al.,^24^ Voss et al.,^28^ Tsai et al.,^25^ Rahe et al.^26^ and Maass et al.^27^ included healthy elderly in their sample, while Baker et al.^12^ and Baker et al.^11^ had elderly with other conditions in their sample such as prediabetic and mild cognitive impairment elderly, respectively. The differences between these samples can represent a confounding factor in the analysis of the studies included in the present review. Despite the low number of studies in this review, one point to be highlighted is that all the experiments were controlled and the samples randomized, which strengthen the results. Nevertheless, two studies included elderly with cognitive pre-dementia conditions and interesting results were found. Thus, verifying the effect of physical exercise on cognitive function and IGF-1 levels in people who have progressive cognitive decline, as in Alzheimer’s Disease, may be a promising field for future studies.

### Training variables, cognitive function and IGF-1

Of the seven studies, five included aerobic exercise in their intervention protocol^11^^,^ 12 ^,^ 26 ^-^ 28 and two had resistance exercise.^24^^,^ 25 Voss et al.^28^ indicated that the effects of exercise type on circulating growth factors (IGF-1, BDNF and VEGF) for the elderly are unknown. On the other hand, aerobic exercise has been the most indicated type of exercise to improve cognition in the elderly population^1^^,^ 29 and this recommendation can explain why most of the papers included in this review - four out of seven - contained aerobic exercise protocols. Regarding their results, the relationship between aerobic exercise and cognitive improvement is inconsistent, since half of the studies found better cognitive performance^11^^,^ 12 after anaerobic training, whereas two studies failed to find significant changes in cognition. However, the different responses might be related to different training variables other than this type of exercise, as well as different outcome measures.

In the first study cited, by Baker et al.,^12^ a cognitive improvement was observed in the executive functions and controlled processes for the aerobic group, and maintenance of IGF-1 levels in the elderly with glucose intolerance was also observed. According to the authors, cognitive improvement can be explained by the physical exercise-induced glucose regulation. In a different study, Baker et al.^11^ observed improvement in executive functions of older women with mild cognitive impairment and, among the older men, there was an increase in IGF-1 levels and better cognitive performance on only one cognitive test (Trails B). The authors claimed that cognitive improvement in older women with mild cognitive impairment has been related to cardiorespiratory fitness increase - peak oxygen uptake increase.

Curiously, for the group that performed aerobic exercise - the type most prevalent in the studies - pre-diabetic elderly was the group that had most notable cognitive improvement and maintenance in IGF-1 levels.^11^ An emerging hypothesis has highlighted diabetes as a preclinical and/or clinical condition for dementia development, especially AD. Researchers indicate that “diabetes type 3” might be linked to the onset of AD symptoms.^31^ In the study of De La Monte et al.,^32^ rats that were subjected to brain insulin deprivation showed plaque formation and neuronal impairment similar to AD. Thus, physical exercise could act on insulin/IGF regulation, preventing the organism from developing diabetes types 2 and/or 3, as well as controlling this condition. Therefore, if we consider pre-diabetes a specific clinical condition for cognitive injury, physical exercise can promote benefits in insulin regulation and sensitivity. This can explain, in part, the clear improvement in the study of Baker et al.^11^ where, despite stable IGF-1 levels, physical exercise promoted significant benefits for cognition.

In the study of Cassilhas et al.,^24^ the control group had lower frequency for the intervention protocol (once a week), compared to the exercise group (50% and 80% of 1RM) that attended sessions 3 times a week. Similar to Cassilhas et al.,^24^ Mass et al.^27^ adopted a protocol in which the exercise and control groups had different weekly frequency: the first group exercised for 40 min, 3 times a week, whereas the control group performed 45 min of muscle relaxation/stretching twice a week. Thus, the conditions provided by the protocols were not similar, causing a bias in the relationship between IGF-1 and cognitive performance. Notably, offering an activity for the control group can be better in ethical and comparison terms than offering nothing, but we would like to call attention to the similar conditions in both groups.

Yau et al.,^33^ in a review study, indicated that there is no unique response in relation to exercise and IGF-1. The authors reported that IGF-1 in adults following acute and chronic exercise was contradictory, where acute exercise induced an increase in IGF-1 levels, whereas chronic exercise induced maintained or decreased levels. Thus, the relationship between IGF-1 and physical exercise can be ambiguous.^33^ Therefore, apparently the same can occur in elderly people, as we found different responses in chronic exercise. However, regarding duration, two studies adopted 12 months of training and the responses in IGF-1 were contradictory.^25^^,^ 28 Further studies on the current topic are warranted.

In a review study, Deslandes et al.^3^ indicated that for inducing IGF-1 increase, resistance exercises seemed to be more effective than aerobic exercises. In the current review, IGF-1 increased more after resistance exercises^24^ than aerobic exercises.^11^^,^ 12 ^,^ 27 ^,^ 28 On the other hand, in a different review published by Kramer and Erickson,^1^ aerobic exercise seemed to exert greater effects on cognitive function than non-aerobic exercise for the elderly.

Regarding type of training, a study in animals that performed both types of training - aerobic and resistance - showed improved cognitive function (memory and spatial learning) and an increase in hippocampal IGF-1 levels, while for peripheral measures, only the resistance group showed improved growth factor.^6^ Thus, it is possible that, apart from the type of training adopted, improvements in cognitive functions occurred through other ways, as indicated by the authors of the cited study. In this case, aerobic exercise increases peripheral and hippocampal BDNF levels, whereas resistance training increases only hippocampal IGF-1 levels. Therefore, IGF-1 peripheral levels can be derived from these types of exercises and muscle growth,^34^ not reflecting central IGF-1 levels. On the other hand, aerobic exercise (moderate treadmill running) in aged rats increased IGF-1, BDNF and NT3 in hippocampus and improved spatial learning and memory, but not in young rats,^35^ showing aging-related differences. Different types of exercise therefore triggered an improvement of different cognitive functions, related to increases or changes in IGF-1 levels or otherwise. The previous study showed that aerobic exercise at moderate intensity could increase peripheral BDNF levels with cognitive function improvement in the elderly.^10^

The intensity of exercise seems to be a key factor to improve cognition. Duzel et al.^36^ indicated mild-to-moderate intensity for preserving and improving cognition in elderly people. Regarding intensity, different protocols analyzed in the present review included mild to high intensity and different intensities can represent a limitation when comparing studies. Only the study of Rahe et al.^26^ did not describe the intensity adopted and how the exercises were offered, since the exercises included cognitive and counseling training. Furthermore, cognitive group, cognitive + exercise training, cognitive + exercise + counseling had similar responses, but only cognitive + exercise training was better than the other two groups in cognitive function gains.

### Brain regions and circulating IGF-1

The study of Maass et al.^27^ employed an interesting brain health measurement: the relationship between changes in IGF-1 and the hippocampus. Although the authors found no improvements in cognitive functions or changes in hippocampus volume after 12 weeks of training, a relationship among circulating IGF-1 changes, hippocampal volume, hippocampal head volume and delayed verbal learning memory test (VLTM) performance was evidenced. Voss et al.^28^ also analyzed the relationship between circulating IGF-1 changes and increases in bilateral parahippocampus and bilateral middle temporal gyrus connectivity. IGF-1 and magnetic resonance imaging (MRI) have both become regarded as important biomarkers related to brain functioning and health.^37^^,^ 38

In the study of Voss et al.,^28^ IGF-1 was correlated with parahippocampal gyrus and middle temporal in elderly who performed aerobic exercises. BDNF and VEGF - factors related to cerebral plasticity - provided a similar response in this gyrus. Thus, the authors deduced that IGF-1 works in conjunction with the factor mentioned previously and can facilitate brain connectivity. In the same study, the intensity used (60-75% of heart rate reserve) for the aerobic training, which may be considered fairly low and, as indicated by the authors, other studies have reported that low intensity may reduce serum IGF-1 levels to 9%.^28^^,^ 39 Attaining a similar result, Maass et al.^27^ confirmed the relationship among IGF-1, hippocampal volume and delayed recall of the VLMT in all participants of their study - training and control groups. Hence, Mass et al.^27^ postulated a relationship between IGF-1 levels and hippocampal volume changes and that hippocampus-dependent memory changes seemed to occur over time independently of exercise.

Unlike in animal studies, it is impossible to measure IGF-1 in the hippocampus. Therefore, neuroimaging plus circulating IGF-1 in human samples can reveal how hormones influence brain structure and functioning. IGF-1 with other peripheral hormones could be considered a part of a functional organization related to environmental and behavioral adaptation.^40^ Also, reduced circulating IGF-1 levels represent a significant factor in the development of cognitive dysfunction.^40^ Thus, measuring circulating IGF-1 along with cognitive functions through neuroimaging and tests are important to understand this relationship and verify response to therapies/interventions. The optimum IGF-1 level and cognition in healthy elderly was also addressed, where a study by Tumati et al.^41^ demonstrated that average values in serum, measured by an immunometric technique, in middle-aged adults and elderly can be better than higher values. Therefore, exercise-related IGF-1 level increase is not a simple question, since there was an ideal level and regulation of it.

### Limitation

Despite the originality of the subject of this paper, some limitation should be outlined. The different protocols adopted in the 7 studies included in this review make comparisons difficult. The exercise type, intensity, volume, frequency and duration could be independent factors affecting exercise response. Human circulating IGF-1 levels provide indirect measures of the nervous system and neuroendocrinology. Therefore, circulating IGF-1 has some limitations and more studies and standard values of this neurotrophic factor are necessary to better understand this subject. Despite the cognitive evaluation, the scales and questionnaires used in most of the studies included can be influenced by several factors, such as mood. Differences among samples, gender-related response, control groups which received stimulation and the number of articles included represent limitations when comparing exercise-induced IGF-1 and cognitive function.

In summary, it was not possible to establish a recommended protocol for exercise type, intensity or duration to promote optimum gain in circulating GF-1 level and increased cognition. However, moderate intensity aerobic training and, moderate and high intensity resistance training may improve circulating IGF-1 and cognition, depending on gender and time duration. Future randomized controlled studies in human samples, including both genders, design variables related to training under similar conditions comparing aerobic and resistance training are needed to clarify this relationship, as well as intensity, duration and volume of exercise.

There seems to be no consensus over cognitive response due to modulated IGF-1 concentrations through physical exercise in the elderly. The different protocols used in the studies, together with the heterogeneous samples, hamper the establishment of peripheral IGF-1 responses to physical exercise related to cognitive functions in the elderly. Thus, this mechanism should be further elucidated and explored.
